# Comparison of Income Eligibility for Medicaid vs Marketplace Coverage for Insurance Enrollment Among Low-Income US Adults

**DOI:** 10.1001/jamahealthforum.2021.0771

**Published:** 2021-06-14

**Authors:** Aditi Bhanja, Dennis Lee, Sarah H. Gordon, Heidi Allen, Benjamin D. Sommers

**Affiliations:** 1 Department of Health Policy and Management, Harvard T.H. Chan School of Public Health, Boston, Massachusetts; 2 Department of Health Policy, Vanderbilt University, Nashville, Tennessee; 3 Department of Health Law, Policy, and Management, Boston University School of Public Health, Boston, Massachusetts; 4 Columbia University School of Social Work, New York, New York; 5 Department of Medicine, Brigham & Women’s Hospital, Boston, Massachusetts

## Abstract

**Importance:**

The Affordable Care Act created 2 new coverage options for uninsured adults: Medicaid expansion, which in most states provides comprehensive coverage without premiums and deductibles; and private marketplace coverage, which requires a premium contribution and cost-sharing, though with generous federal subsidies at lower incomes. How enrollment rates compare in the marketplace vs Medicaid is an important policy question as states continue to weigh alternative coverage options such as Medicaid buy-in programs, enrolling Medicaid-eligible populations into marketplace plans, or creating a public option.

**Objective:**

To assess the association between income eligibility for Medicaid vs marketplace coverage and insurance enrollment among low-income adults in Colorado.

**Design, Setting, and Participants:**

Using 2014 and 2015 all-payer claims data from Colorado and detailed income eligibility information, we used a regression discontinuity design to assess the difference in Medicaid and marketplace enrollment just below and just above 138% of the federal poverty level (FPL), the eligibility threshold between the 2 programs. The sample included nonpregnant adults aged 19 to 64 years with incomes between 75% to 400% FPL. We stratified our analysis by age, sex, chronic condition status, and urban vs rural residence. Analysis was conducted from January to October 2020.

**Main Outcome and Measures:**

The main outcome was total enrollment in either Medicaid or marketplace coverage during marketplace’s Open Enrollment period. Income-based health insurance eligibility was assessed as a percentage of FPL at the time of initial application for coverage.

**Results:**

The primary analytical sample included 32 091 enrollees in 2014 and 55 451 in 2015, with incomes ranging from 120% to 156% FPL. Most enrollees were women (59.26% in 2014, 59.20% in 2015), resided in urban areas (70.36% in 2014, 73.08% in 2015), and had no chronic conditions (74.66% in 2014, 76.11% in 2015). For age, in 2014 and 2015, respectively, 13.22% and 13.93% were aged 19 to 25 years, 27.85% and 28.54% were aged 26 to 34 years, 23.58% and 24.34% were aged 35 to 44 years, 18.35% and 17.75% were aged 45 to 54 years, and 17.00% and 15.44% were aged 55 to 64 years. Marketplace enrollment was 81.3% (95% CI, −86.0% to −75.0%) lower than Medicaid enrollment in 2014 and 88.6% (95% CI, −90.8% to −86.0%) lower in 2015 among those close to the 138% FPL eligibility threshold. The drop-off in marketplace enrollment was largest among younger adults, aged 26 to 34 and 35 to 44 years: relative drop off −88.7% (95% CI, −93.3% to −80.8%) and −87.8% (95% CI, −90.8% to −83.9%) in 2014, and relative drop off –91.9% (95% CI, −94.5% to −87.9%) and −93.0% (95% CI, −94.5% to −91.1%) in 2015, respectively.

**Conclusions and Relevance:**

In this cross-sectional study using a regression-discontinuity analysis, meaningful gaps in insurance enrollment may have existed for those with incomes just above the eligibility threshold for Medicaid expansion, especially among younger adults. Policies expanding Medicaid income eligibility or zero-dollar premium marketplace plans are likely to be more effective at inducing enrollment than subsidized private plans with premium requirements.

## Key Points

**Question:**

How did health insurance enrollment rates compare for Medicaid vs subsidized marketplace coverage among low-income adults in Colorado 2014 to 2015?

**Findings:**

In this cross-sectional study with a regression discontinuity analysis of 87 542 US adult enrollees in Medicaid and private insurance, marketplace enrollment was 81.3% lower than Medicaid enrollment in 2014 and 88.6% lower in 2015 among those close to the eligibility threshold. The drop-off in marketplace enrollment was largest among younger adults.

**Meaning:**

Substantial gaps in publicly subsidized private coverage may have existed for those with incomes just beyond the reach of Medicaid expansion, especially among younger adults.

## Teaser

This cross-sectional study assesses the association between income eligibility for Medicaid vs marketplace coverage and insurance enrollment among adults with low income in Colorado.

## Introduction

The Affordable Care Act (ACA) created 2 important coverage options for uninsured adults beginning in 2014.^1^ Individuals with incomes at or below 138% of the federal poverty level (FPL) are eligible for Medicaid expansion in participating states, which provides comprehensive coverage without premiums and minimal cost sharing. Individuals with incomes between 138% and 400% FPL can purchase private marketplace coverage, which requires a premium contribution and cost-sharing, but includes income-based tax credits and cost-sharing subsidies from the federal government.

Since then, 39 states (including the District of Columbia) have expanded Medicaid and almost 20 million low-income adults have gained health insurance.^1,2^ For those with incomes above the expanded Medicaid eligibility threshold, marketplace coverage has also decreased the uninsured rate and improved access to care for low-income adults without employer-sponsored insurance (ESI).^3^

Despite these coverage gains, almost 30 million people remained uninsured in 2019, and over 7 million of them were likely eligible for marketplace tax credits.^4^ Research suggests that lack of affordability may deter enrollment for those eligible for marketplace coverage.^5^ Although premiums for low-income individuals are subsidized,^6^ marketplace plans often include high deductibles and point-of-care cost sharing. Previous studies of insurance indicate modest premiums and higher cost sharing are barriers to coverage among low-income individuals.^7,8^ Various subgroups may differ in their price sensitivity for coverage, with low-income individuals, younger adults, and the self employed potentially more sensitive to premiums.^9,10^ This may be compounded by administrative hurdles to enrolling in marketplace coverage, and confusion related to ACA complexity and misinformation campaigns.^11,12^

Recent federal and state proposals to address these gaps included options, such as improving private coverage and marketplace affordability through increased subsidies and reduced cost sharing^13^; expanding Medicaid’s low-cost comprehensive coverage with a subsidized premium buy-in; or replacing eligibility for the Medicaid expansion in some populations with private insurance.^14,15^ Evidence on factors driving differential enrollment rates could provide key insights on ways to insure those with the greatest need.

In this Colorado-based analysis, we compare individuals near the eligibility threshold of 138% of FPL to assess the association between eligibility for Medicaid vs Marketplace coverage and public insurance enrollment among low-income adults and whether enrollment differs by age, sex, chronic condition status, or residence. With this data set, we previously compared health care costs, quality, and utilization between Medicaid and Marketplace enrollees across the expanded Medicaid eligibility threshold. We found modest differences in quality, significantly higher costs among Marketplace enrollees, and higher emergency department visits among Medicaid enrollees.^16^ In this study, we assess an important question that was not explored in our prior research, the overall likelihood of enrollment in these 2 types of coverage.

## Methods

### Data and Study Design

Using all-payer claims data (APCD) from Colorado and detailed income eligibility information provided by the state’s Medicaid agency and marketplace, we assessed the difference between Medicaid and Marketplace enrollment just above and below the 138% FPL cutoff, using a sharp regression discontinuity design (RD) (eMethods and eTable1 in the Supplement). This project was approved by the Harvard T. H. Chan School of Public Health’s institutional review board. As this study used preexisting, deidentified secondary data for analysis, informed consent was waived. This study also followed the Strengthening the Reporting of Observational Studies in Epidemiology (STROBE) reporting guidelines.

We limited the sample to nonpregnant adults aged 19 to 64 years with incomes between 75% to 400% FPL enrolled in Medicaid or marketplace coverage for at least 1 month during 2014 or 2015 (eMethods in the Supplement).

The study outcome was the number of persons enrolled in Medicaid or marketplace insurance in each FPL bin. To account for differences in when patients can enroll in Medicaid compared with the marketplace, we limited the outcome to coverage initiated during the ACA’s open enrollment periods (January 1, 2014, to April 15, 2014, and January 1, 2015, to March 3, 2015). Because most individuals below the 138% FPL threshold were covered by Medicaid and those above, by marketplace, we compare the enrollment differences across the threshold by coverage rather than income. The analysis was conducted from January 2020 to October 2020, using STATA statistical software (version 14.0; StataCorp LLC).

### Statistical Analysis

We conducted descriptive analyses of our sample by age, sex, chronic condition status (any Elixhauser-defined condition identified through *International Classification of Diseases, Ninth Revision, Clinical Modification and (*ICD-9*)* and *ICD-10* codes) and urban vs rural residence using scatterplots of enrollment in each bin of family income as a percentage of the FPL. We analyzed enrollment using 2 sets of RD models (eMethods in the Supplement).

Our first set of models employed a nonparametric approach in which we narrowed the sample around the 138% threshold and specified a local linear regression. The key variable of interest was a binary indicator for income greater than 138% of FPL, signaling the discontinuity in coverage along income as a function of the key Medicaid-to-marketplace eligibility transition. We tested this approach using various bandwidths, including the optimal bandwidth approach.^17^ These nonparametric models used a triangular kernel weighting approach, in which values closest to 138% of FPL were weighted more heavily.

Our second set of models employed a parametric approach that used the full income range of the sample (75%-400% of FPL) and modeled the association between income and enrollment as a polynomial, allowing for linear, quadratic, and cubic terms. As in the local linear model, we allowed this association to vary above and below the 138% threshold.

We use a generalized linear model with a negative binomial distribution and log link, and present estimated coefficients as incidence rate ratios, which allowed for direct comparisons of the relative reduction in enrollment between subgroups. We repeated stratified analyses for year, age, sex, chronic condition status, and urban vs rural residence. Using simultaneous covariance matrices with cross-model χ^2^ analyses (adjusted Wald tests in the local linear models), we tested for significant differences between enrollment estimates for each subgroup.

We also conducted 2 sensitivity analyses (1) replicating our methods using linear regression models; and (2) examining any discontinuities in income for the Colorado population using data from the American Community Survey (ACS, eMethods in the Supplement).

## Results

The primary analytical sample for the local linear model included 32 091 enrollees in 2014 and 55 451 in 2015, with incomes ranging from 120% to 156% FPL. Most enrollees were women (59.26% in 2014, 59.20% in 2015), resided in urban areas (70.36% in 2014, 73.08% in 2015) and had no chronic conditions (74.66% in 2014, 76.11% in 2015). For age, in 2014 and 2015, respectively, 13.22% and 13.93% were aged 19 to 25 years, 27.85% and 28.54% were aged 26 to 34 years, 23.58% and 24.34% were aged 35 to 44 years, 18.35% and 17.75% were aged 45 to 54 years, and 17.00% and 15.44% were aged 55 to 64 years (Table 1).

**Table 1.** Sample Characteristics, Optimal Bandwidth, and Total Sample, 2014 and 2015^a^

Characteristic	No. (%)			
	Optimal bandwidth^b^		Total sample	
	2014	2015	2014	2015
FPL, %	120-156		75-400	
Open enrollment	32 091	55 481	142 176	213 561
Age, y				
19-25	4242 (13.22)	7727 (13.93)	18 075 (12.71)	28 637 (13.41)
26-34	8936 (27.85)	15 832 (28.54)	38 273 (26.92)	59 199 (27.72)
35-44	7568 (23.58)	13 506 (24.34)	33 099 (23.28)	51 337 (24.04)
45-54	5889 (18.35)	9848 (17.75)	25 723 (18.09)	37 842 (17.72)
55-64	5456 (17.00)	8568 (15.44)	27 006 (18.99)	36 546 (17.11)
Sex				
Female	19 017 (59.26)	32 843 (59.20)	86 124 (60.58)	128 721 (60.27)
Male	13 063 (40.71)	22 612 (40.76)	55 999 (39.39)	84 767 (39.69)
Chronic condition				
Any	8131 (25.34)	13 257 (23.89)	38 090 (26.79)	52 408 (24.54)
None	23 960 (74.66)	42 224 (76.11)	104 086 (73.21)	161 153 (75.46)
Residence				
Urban	22 578 (70.36)	40 544 (73.08)	98 594 (69.35)	154 929 (72.55)
Rural	3295 (10.27)	4941 (8.91)	14 894 (10.48)	19 924 (9.33)

Abbreviation: FPL, federal poverty level.

^a^ Source: Analysis of Colorado all-payer claims database from 2014-2015.

^b^ The optimal bandwidth, here defined as 120% to 156% FPL, was set using the STATA SE 14 MSE−optimal bandwidth selector for the RD treatment effect estimator e(h_mserd), defined by the command rdbwselect using the 2014 enrollment outcome.

**Figure 1.**
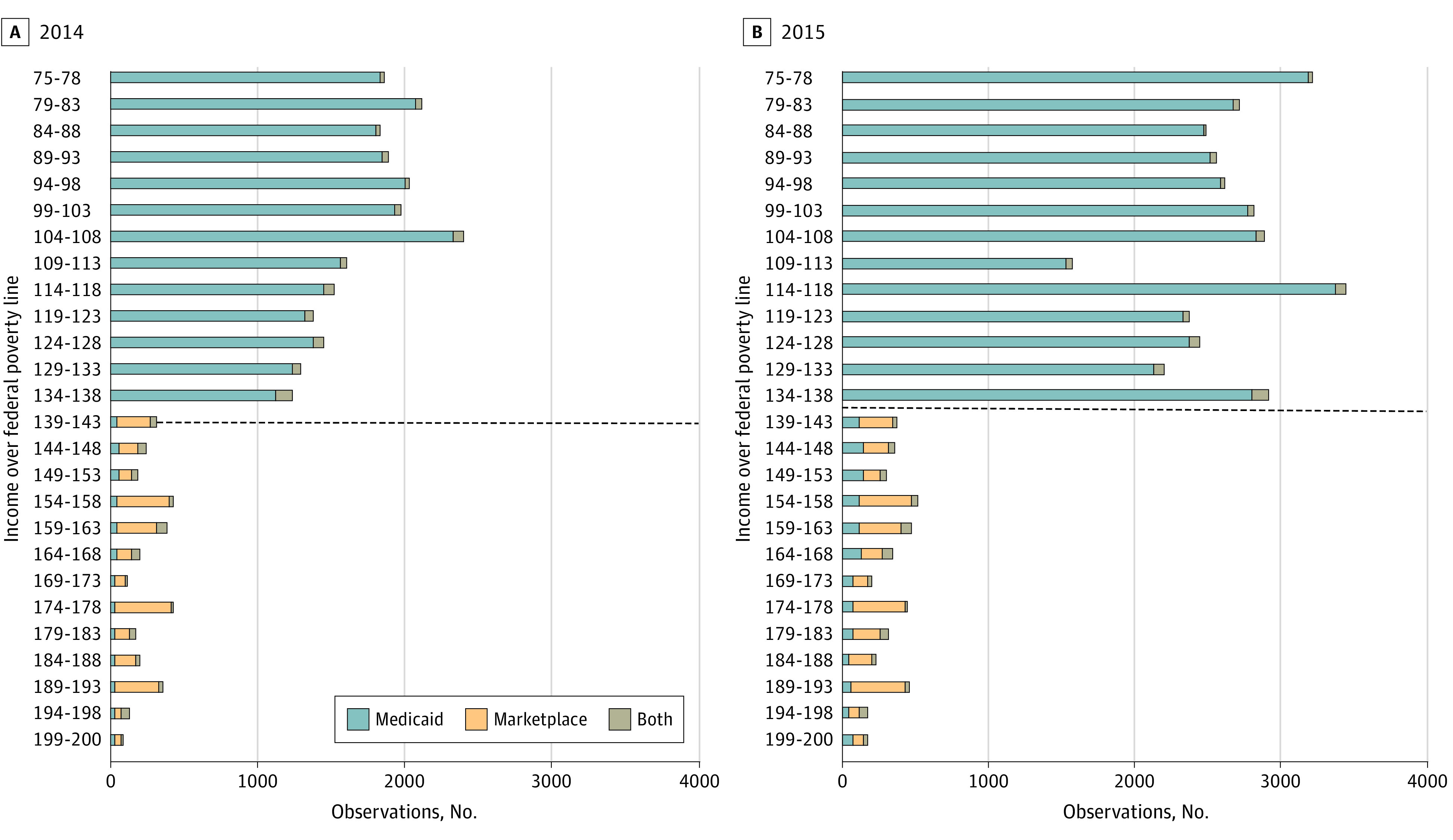
Enrollment in Medicaid and Marketplace Coverage among Nonelderly Adults by Income, 2014 and2015 The sample was limited to nondisabled and nonpregnant adults aged 19-64 whose Medicaid or marketplace coverage became active during the ACA’s open enrollment period (January 1, 2014, to April 15, 2014, and January 1, 2015, to March 3, 2015). Individuals are classified based on their initial income at the time of application, but owing to income changes during the year, some individuals end up enrolled in both programs and/or the program other than their initial eligibility determination. Source: Analysis of Colorado all-payer claims database from 2014 to 2015.

Figure 1 presents the total enrollment by coverage type in each bin of FPL for the sample, 75% to 200% FPL, which shows a sharp drop-off in the number of individuals enrolled in ACA-related coverage above 138% FPL in 2014 and 2015.

Our primary regression results are from the local linear model using the optimal bandwidth, with incomes between 120% and 156% FPL. The relative drop-off in total enrollment in ACA-related coverage between 138% and 139% FPL was −81.3% in 2014 (95% CI, −86.0% to −75.0%) and −88.6% in 2015 (95% CI, −90.8% to −86.0%) in the local linear model (Table 2).

**Table 2.** Enrollment Drop-Off for Adults Eligible for Marketplace Coverage (>138% of FPL), Compared With Medicaid (≤138% of FPL)^a^

Variable	Year	
	2014	2015
Average Medicaid enrollment per percentage point of FPL (133-138 FPL)	1424 (353.33)	2882 (383.83)
Relative enrollment change, Marketplace vs Medicaid, % (95% CI)	−81.3 (−86.0 to −75.0)	−88.6 (−90.8 to −86.0)
*P* value	<.001	<.001

Abbreviation: FPL, federal poverty level.

^a^ Source: Analysis of Colorado all-payer claims database from 2014 to 2015.

Change in enrollment comes from the regression discontinuity coefficient as an incident rate ratio from a local linear model using the optimal bandwidth, here defined as 120% to 156% FPL.

Sample was limited to nondisabled and nonpregnant adults aged 19 to 64 years whose Medicaid or marketplace coverage became active during the ACA’s open enrollment period (January 1, 2014, to April 15, 2014, and January 1, 2015, to March 3, 2015).

Subgroup analyses also showed significant drop-offs in enrollment for those above 138% FPL.

**Figure 2.**
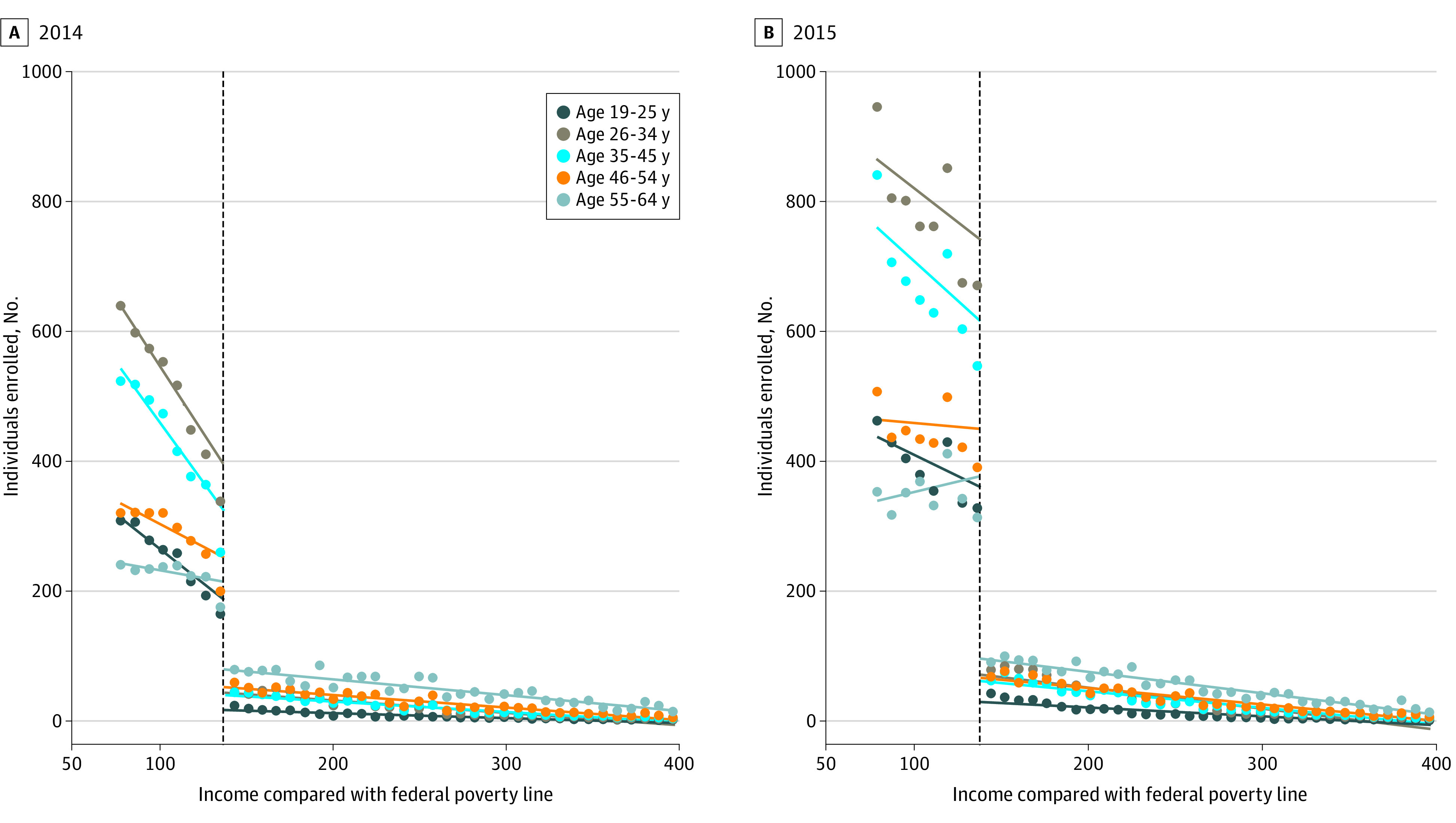
Number of Medicaid and Marketplace Enrollees By Age Group and Income, 2014 and 2015 Sample was limited to nondisabled and nonpregnant adults aged 19 to 64 years whose Medicaid or Marketplace coverage became active during the ACA’s open enrollment period (January 1, 2014, to April 15, 2014, and January 1, 2015, to March 3, 2015). Source: Analysis of Colorado all-payer claims database from 2014 to 2015.

In both years, differences in enrollment varied most widely by age group. Younger enrollees—those aged 26 to 34 years and 35 to 44 years—had the largest enrollment drop-offs: −88.7% (95% CI, −93.3% to −80.8%) and −87.8% (95% CI, −90.8% to −83.9%) in 2014, and −91.9% (95% CI, −94.5% to −87.9) and −93.0% (95% CI, −94.5% to −91.1%) in 2015, respectively (Figure 2) (Table 3). Enrollees aged 55 to 64 years had the smallest enrollment drop-offs among any subgroups, −56.4% (95% CI, −70.1% to −36.3%) in 2014 and −75.3% (95% CI, −81.5% to −66.7%) in 2015. The differences by age group were statistically significant in both years (*P* < .001 for all age groups compared with those aged 55-64 years).

**Table 3.** Enrollment Drop-Off for Adults Eligible for Marketplace Coverage, Compared With Medicaid, by Subgroup^a^

Characteristic	2014		2015	
	Relative enrollment drop-off, % (95% CI)	*P* value	Relative enrollment drop-off, % (95% CI)	*P* value
Age, y				
19-25	−86.7 (−91.3 to −79.6)	<.001	−88.6 (−90.6 to −86.0)	<.001
26-34	−88.7 (−93.3 to −80.8)	<.001	−91.9 (−94.5 to −87.9)	<.001
35-44	−87.8 (−90.8 to −83.9)	<.001	−93.0 (−94.5 to −91.1)	<.001
45-54	−74.6 (−79.6 to −68.3)	<.001	−86.6 (−89.1 to −83.4)	<.001
55-64^b^	−56.4 (−70.1 to −36.3)	1 [Reference]	−75.3 (−81.5 to −66.7)	1 [Reference]
Sex				
Female	−81.6 (−85.9 to −76.1)	.57	−89.5 (−91.6 to −86.9)	<.001
Male^b^	−80.7 (−86.6 to −72.1)	1 [Reference]	−87.3 (−89.8 to −84.2)	1 [Reference]
Chronic condition				
Any	−79.3 (−84.9 to −71.5)	.14	−88.5 (−90.8 to −85.3)	.65
None^b^	−81.9 (−86.6 to −75.4)	1 [Reference]	−88.7 (−90.9 to −86.0)	1 [Reference]
Residence				
Urban	−81.3 (−85.9 to −75.1)	.24	−89.1 (−91.3 to −86.1)	.016
Rural^b^	−82.0 (−85.1 to −70.4)	1 [Reference]	−83.1 (−87.6 to −76.7)	1 [Reference]

^a^ Source: Analysis of Colorado all-payer claims database from 2014-2015.

All group coefficients differ significantly from zero at *P* < .001.

“Change in enrollment” comes from the regression discontinuity coefficient from a local linear model using the optimal bandwidth, here defined as 120-156% FPL.

Sample was limited to nondisabled and nonpregnant adults aged 19 to 64 years whose Medicaid or marketplace coverage became active during the ACA’s open enrollment period (January 1, 2014, to April 15, 2014, and January 1, 2015, to March 3, 2015).

^b^ Reference group for adjusted Wald Test Model comparisons.

The relative drop-off in ACA-related enrollment above 138% FPL was slightly larger for women than for men in both years, −81.6% (95% CI, −85.9% to −76.1%) in 2014 and −89.5% (95% CI, −91.6% to −86.9%) in 2015 for female enrollees, compared with −80.7% (95% CI, −86.6% to −72.1%) in 2014 and −87.3% (95% CI, −89.8% to −84.2%) in 2015 for male enrollees (Table 3) (eFigure 1 in the Supplement). These differences were statistically significant in 2015 (*P* < .001), but not in 2014 (*P* = .57).

In 2014, urban and rural residents experienced similar drop-offs in enrollment above 138% FPL, −81.3% (95% CI, −85.9% to −75.1%) and −82.0% (95% CI, −85.1% to −70.4%), respectively (*P* = .24), but the gap widened in 2015, with drop-offs of −89.1% (95% CI, −91.3% to −86.1%) for urban residents and −83.1% (95% CI, −87.6% to −76.7%) for rural residents (Table 3) (eFigure 2 in the Supplement), which was statistically significant in 2015 (*P* = .02).

Those with chronic conditions showed slightly lower drop-offs in enrollment than those without chronic conditions in both years, −79.3% (95% CI, −84.9% to −71.5%) compared with −81.9% (95% CI, −86.6% to −75.4%) in 2014 (*P* = .14) and −88.5% (95% CI, −90.8% to −85.3%) compared with −88.7% (−90.9% to −86.0%) in 2015 (*P* = .65), respectively, but these differences were not statistically significant (Table 3) (eFigure 3 in the Supplement).

Results for parametric and nonparametric models (eTable 2, eTable 3, eTable 4, eTable 5, and eTable 6 in the Supplement) were similar to our main model, showing large ACA enrollment drop-offs above 138% FPL for all subgroups, with the largest drop-offs among younger adults. Our linear regression coefficients showed comparable drop-offs in enrollment, calculated as a proportion of the average enrollment between 133% to 138% FPL, −76.4% in 2014 (n = 1424; standard deviation [SD],353.33) and −84.7% in 2015 (n = 2882; SD, 383.83) (eTable 7 in the Supplement).

Analysis of the ACS indicates no discontinuity in the income distribution in Colorado at the 138% FPL eligibility threshold in 2014 or 2015 (eFigure 4 in the Supplement).

## Discussion

In this regression discontinuity analysis of marketplace and Medicaid health insurance enrollment among low-income adults in Colorado, we find that marketplace enrollment was 81.3% lower than Medicaid enrollment in 2014 and 88.6% lower in 2015, just above the 138% FPL income cutoff. This sharp drop-off in enrollment in ACA coverage suggests potential coverage gaps for those with incomes just above the Medicaid eligibility range.

Many factors potentially contribute to these differential coverage rates, such as difficulty affording marketplace plans even with substantial premium subsidies, administrative or informational barriers to enrolling in marketplace coverage, and the ACA exclusion of those with an affordable ESI offer from receipt of marketplace subsidies.

Lower-income adults have the highest uninsurance rates of any group in the US, and costs of coverage are cited as the most common reason for lacking insurance.^1^ In 1 national survey, 57% of individuals who visited a marketplace to shop for plans, but did not end up enrolling, named affordability concerns.^18^ As for those who do enroll in coverage, a longitudinal study using Colorado’s APCD found that 1 in 4 marketplace enrollees dropped out before the end of the year; those who received greater financial assistance disenrolled at lower rates.^19^ Our results suggest that even these highly subsidized premiums may still be a substantial barrier to enrollment. Although lower-cost plans are available for those electing for less generous coverage,^20^ bronze plans with high deductibles may also dissuade some enrollees, compared with Medicaid.

Overall, only 37% of Coloradoans eligible for financial assistance enrolled in marketplace coverage in 2019,^21^ signaling more nuance to the issue of affordability. High deductibles, higher cost sharing for high-generosity coverage, even political disinformation related to marketplace costs, may all reduce marketplace enrollment among low-income populations.

Regarding administrative hurdles, research has found that lag times, poor communication about eligibility, difficulty navigating online information, and complexity of materials for plan selection were among several reasons why eligible individuals had forgone marketplace enrollment.^22^ Although research shows that similar administrative barriers are also associated with Medicaid enrollment,^23^ it is possible that they are less onerous in a program designed to cover low-income populations^24^ and without the added complexity of choosing a plan. For low-income adults who are eligible for subsidies, studies suggest low-touch, low-cost interventions that mitigate these barriers may improve marketplace enrollment.^11^^,^^25,26^

The drop-off in marketplace enrollment relative to Medicaid could also be related to the ACA’s exclusion of individuals with an affordable ESI offer from marketplace subsidies. Under the ACA, ESI offers are deemed affordable if the lowest-cost individual plan is at or under 9.78% of the total household income. However, this is unlikely to explain enrollment drop-offs of the magnitude we observed because ESI is simply not that common in this income range; only 28% of nonelderly Coloradoans with incomes below 200% FPL were covered under ESI in 2014 to 2015.^27^

### At-Risk Groups for Low Marketplace Enrollment

In our sample, the enrollment drop-off above 138% FPL was disproportionately higher among younger adults. Those aged 26 to 44 years saw the largest enrollment differences at the eligibility threshold, around 90% lower in marketplace coverage compared with Medicaid. Comparatively, those aged 55 to 64 had a 56% to 75% enrollment drop-off. The disparity by age could reflect different levels of priority on the perceived need for health insurance. Another factor driving age group differences may be variation in affordability of premiums. A comparison of the lowest-cost bronze plans across all Medicaid-expanded states showed that young and middle-age adults paid a higher percentage of their income in after-subsidy premiums than their older counterparts at 200% FPL.^28^ Prior studies and our findings raise concerns about the marketplace risk pool; some analysts have proposed increased premium subsidies for those aged 35 to 44 years to curtail adverse selection.^29,30^

We also found smaller yet significant differences in enrollment by sex and residence, with larger enrollment drop-offs in 2015 among women and urban residents than men and rural residents, respectively. Prior research shows that women experienced substantial coverage gains under the ACA, and it is unclear what may be driving the larger enrollment drop-off for women in 2015.^31^ As for the disparities by urban or rural status in 2015, Colorado dedicated more marketing efforts in rural areas to increase uptake for low-enrollment populations,^32^ which may have driven the smaller drop-off in enrollment.

### Limitations

Our study has several limitations. First, we only captured individuals enrolled in Medicaid or marketplace coverage, meaning that we cannot directly observe whether the enrollment drop-off corresponded to an increase in uninsured rates, a change in the denominator of individuals at a particular income level, or different rates in alternative coverage such as ESI. Using ACS data, we found no evidence of a sudden shift in the population denominator at or near the 138% FPL cutoff. Nonetheless, we were unable to assess the degree to which ESI enrollment or exclusion from ACA coverage owing to an affordable ESI offer explains the enrollment differences between Medicaid and marketplace coverage in these data. Our findings are consistent with prior research on marketplace take-up and higher rates of uninsurance in lower-income and younger populations, in Colorado and nationally.^1,21^ We also only observed effectuated enrollment, which did not capture individuals who signed up for marketplace coverage but did not pay their first premium.

Second, race and ethnicity were poorly and differentially captured in our Medicaid and marketplace data, precluding any assessment of racial and ethnic subgroups. Future research using other data sources is critical to examining potential racial disparities in this context. In addition, our data includes 100% of Medicaid enrollment, but approximately 83% of Colorado’s marketplace (eMethods in the Supplement). Some of our observed drop-off is likely attributable to these missing data.

Third, our subgroup analyses examined adults based on their underlying health status, but we were limited to doing so based on diagnosis codes present in the claims database. This may have underestimated the true incidence of chronic conditions in our sample (and may do so differentially for public vs private insurance) if some patients with these conditions did not obtain medical care resulting in a billed diagnosis. Similarly, information on substance abuse disorders in the database was limited owing to federal privacy laws, which also may have contributed to an underestimate of chronic conditions in our study.

Finally, our sample includes Colorado residents only, which may limit the generalizability of these findings to other states.

### Policy Implications

These findings indicate that low-income adults in Colorado appear over 80% less likely to enroll in ACA-related health insurance coverage when the option is marketplace insurance rather than Medicaid. As policymakers consider various approaches to enrolling more individuals in insurance, zero-premium comprehensive coverage in Medicaid offers a substantial advantage in enrollment compared with highly subsidized private insurance requiring a premium. This suggests the proposals by states, such as Massachusetts, to move some Medicaid beneficiaries into private coverage, could produce large losses in insurance.^14^ However, zero-premium marketplace plans, such as the public option proposed by policymakers for nonexpansion states, have been shown to improve marketplace enrollment, especially for low-income adults.^33^ Automatic enrollment, another part of this proposal, would likely have similar effects^34^ and may promote continuity of coverage^35,36^; together, these options could significantly improve effectuated enrollment for this population by removing both administrative and financial barriers to coverage.^37^

Meanwhile, models that offer coverage to those above the 138% income cutoff in programs more similar to Medicaid, such as the Basic Health Program in New York and Minnesota, may have greater success at covering uninsured populations. Out-of-pocket costs in marketplace are substantially higher than in Medicaid, which may lead to disenrollment.^16,19^ Other policymakers have proposed benchmarking marketplace premium tax credits to gold plans to increase the overall subsidy, which could offer similar affordability improvements by reducing premiums on plans with higher cost-sharing assistance.

Amidst the coronavirus 2019 (COVID-19) pandemic, coverage gaps for low-income adults are increasingly relevant for policymakers. Between January and May 2020, more than 40 million individuals lost their jobs,^38^ which for many also meant a loss of their ESI. Although a significant proportion may obtain coverage from Medicaid or the marketplaces, with Medicaid enrolling most, studies have predicted anywhere from 3.5 to 7 million will be left without insurance.^39,40^ Though unemployment rates are changing with different COVID-19 restrictions, layoffs continue to disproportionately affect younger adults, with those aged 25 to 34 years experiencing increases in unemployment at roughly 1.5 times the rate of those aged 45 years and older.^41^ In the context of these findings, newly unemployed younger low-income adults may be at high risk for becoming uninsured if they do not qualify for Medicaid’s premium-free coverage and if this pattern of discontinuity persists.

## Conclusions

In this cross-sectional study using a regression discontinuity analysis, our findings showed that significant enrollment differences in public health insurance existed for Coloradoans with incomes just above the expanded Medicaid eligibility threshold in 2014 and 2015, particularly for younger adults. Policies extending Medicaid or zero-dollar premium plans are likely to be more effective at inducing enrollment for this population than subsidized private plans with premium requirements. Policymakers should be aware of enrollment differences between Medicaid and subsidized private insurance and focus on implementing changes that could substantially increase enrollment and curtail age disparities for low-income adults.

## Notes

Supplement. eMethods. eTable 1. Marketplace Enrollment by Plan eFigure 1. Number of Medicaid/Marketplace Enrollees By Gender and Income, 75-400% FPL, 2014 and 2015 eFigure 2. Number of Medicaid/Marketplace Enrollees By Residence, Urban vs. Rural, and Income, 75-400% FPL, 2014 and 2015 eFigure 3. Number of Medicaid/Marketplace Enrollees By Chronic Condition Status and Income, 75-400% FPL, 2014 and 2015 eTable 2. Open Enrollment, Full Linear, Quadratic, Cubic, and Local Linear Regression Discontinuity eTable 3. Open Enrollment by Age, Full Linear, Quadratic, Cubic, and Local Linear Regression Discontinuity, 2014 eTable 4. Open Enrollment by Age, Full Linear, Quadratic, Cubic, and Local Linear Regression Discontinuity, 2015 eTable 5. Open Enrollment by Sex, Chronic Condition and Residence, Full Linear, Quadratic, Cubic, and Local Linear Regression Discontinuity, 2014 eTable 6. Open Enrollment by Sex, Chronic Condition and Residence, Full Linear, Quadratic, Cubic, and Local Linear Regression Discontinuity, 2015 eTable 7. Open Enrollment, Full Linear, Quadratic Cubic, and Local Linear Regression Discontinuity Sensitivity Analysis eFigure 4. Population by Income, Colorado American Community Survey, 2014 -2015
