# Supplementary material for: Comparison of Income Eligibility for Medicaid vs Marketplace Coverage for Insurance Enrollment Among Low-Income US Adults
Source: JAMA Health Forum. 2021 Jun 14;2(6):e210771. doi: 10.1001/jamahealthforum.2021.0771 (PMC8796906; doi:10.1001/jamahealthforum.2021.0771)
Supplement: Supplement. — eMethods. eTable 1. Marketplace Enrollment by Plan eFigure 1. Number of Medicaid/Marketplace Enrollees By Gender and Income, 75-400% FPL, 2014 and 2015 eFigure 2. Number of Medicaid/Marketplace Enrollees By Residence, Urban vs. Rural, and Income, 75-400% FPL, 2014 and 2015 eFigure 3. Number of Medicaid/Marketplace Enrollees By Chronic Condition Status and Income, 75-400% FPL, 2014 and 2015 eTable 2. Open Enrollment, Full Linear, Quadratic, Cubic, and Local Linear Regression Discontinuity eTable 3. Open Enrollment by Age, Full Linear, Quadratic, Cubic, and Local Linear Regression Discontinuity, 2014 eTable 4. Open Enrollment by Age, Full Linear, Quadratic, Cubic, and Local Linear Regression Discontinuity, 2015 eTable 5. Open Enrollment by Sex, Chronic Condition and Residence, Full Linear, Quadratic, Cubic, and Local Linear Regression Discontinuity, 2014 eTable 6. Open Enrollment by Sex, Chronic Condition and Residence, Full Linear, Quadratic, Cubic, and Local Linear Regression Discontinuity, 2015 eTable 7. Open Enrollment, Full Linear, Quadratic Cubic, and Local Linear Regression Discontinuity Sensitivity Analysis eFigure 4. Population by Income, Colorado American Community Survey, 2014 -2015 [file jamahealthforum-e210771-s001.pdf]

Supplemental Online Content

Bhanja A, Lee D, Gordon SH, Allen H, Sommers BD. Comparison of income eligibility for Medicaid vs marketplace coverage for insurance enrollment among low-income US adults. *JAMA Health Forum*. 2021;2(6):e210771.
doi:10.1001/jamahealthforum.2021.0771

eMethods.
eTable 1. Marketplace Enrollment by Plan
eFigure 1. Number of Medicaid/Marketplace Enrollees By Gender and Income, 75-400% FPL, 2014 and 2015
eFigure 2. Number of Medicaid/Marketplace Enrollees By Residence, Urban vs. Rural, and Income, 75-400% FPL, 2014 and 2015
eFigure 3. Number of Medicaid/Marketplace Enrollees By Chronic Condition Status and Income, 75-400% FPL, 2014 and 2015
eTable 2. Open Enrollment, Full Linear, Quadratic, Cubic, and Local Linear Regression Discontinuity
eTable 3. Open Enrollment by Age, Full Linear, Quadratic, Cubic, and Local Linear Regression Discontinuity, 2014
eTable 4. Open Enrollment by Age, Full Linear, Quadratic, Cubic, and Local Linear Regression Discontinuity, 2015
eTable 5. Open Enrollment by Sex, Chronic Condition and Residence, Full Linear, Quadratic, Cubic, and Local Linear Regression Discontinuity, 2014
eTable 6. Open Enrollment by Sex, Chronic Condition and Residence, Full Linear, Quadratic, Cubic, and Local Linear Regression Discontinuity, 2015
eTable 7. Open Enrollment, Full Linear, Quadratic Cubic, and Local Linear Regression Discontinuity Sensitivity Analysis
eFigure 4. Population by Income, Colorado American Community Survey, 2014 -2015

This supplemental material has been provided by the authors to give readers additional information about their work.

© 2021 Bhanja A et al. *JAMA Health Forum*.

eMethods

Dataset Construction

The Colorado APCD is an analytical dataset constructed by the Center for Improving Value in Health Care (CIVHC). With CIVHC as our coordinating partner, we merged the APCD with eligibility and income files provided by the Colorado Department of Health Care Policy and Financing (HCPF, which oversees Health First Colorado (Colorado's Medicaid Program) and the Connect for Health Colorado (C4HCO, the state's health insurance Marketplace). [1] Our analysis used the enrollment files, which indicate the months of coverage and type of coverage for each individual in Medicaid or Marketplace insurance, as well as demographic information such as age, sex, and urban vs. rural residence (based on 3-digit zip codes). Notably, race and ethnicity are missing for many observations and are inconsistently recorded in both programs, precluding any analysis of subgroups by race/ethnicity. The APCD also includes outpatient and inpatient claims and diagnosis codes (ICD 9/10), which we used to identify individuals with chronic conditions and pregnant women. Chronic conditions were defined by those included in the Elixhauser comorbidity index.[2]

Sample Representation

Over the study period, 11 health insurance plans operated in the Colorado Marketplace, excluding dental insurance plans. The CO APCD includes six of these, while BEST Life and Health Insurance Company left the Marketplace in 2014 and ceased submitting claims and Colorado HealthOP left in 2016 but does not appear in the data. We assessed the share of Marketplace enrollees captured by our dataset, by comparing the sample size in our data files to the publicly-reported totals of individuals in the Colorado Marketplace. Our 2014 sample contained 62,336 Marketplace individuals with income data and FPL < 400, compared to approximately 76,110 individuals who enrolled in C4CHO and received subsidies in 2014 (59% of the 129,000 reported by C4HCO's official statistics.[3] For 2015, the comparable numbers were 64,165 Marketplace individuals with income data and FPL < 400 and 76,700 who enrolled in C4CHO and received subsidies in 2015.[4] This indicates our sample contains data for 81.9% and 83.7% of the state's subsidized Marketplace population for 2014 and 2015, respectively.

eTable 1. Marketplace Enrollment by Plan

	C4HCO		
**Enrollments**	2014	2015	Notes
Access Health Colorado	154	792	
Anthem Blue Cross and Blue Shield - HMO Colorado (3)	18,700	14,496	
BEST Life and Health Insurance Company (1)	321	-	2015 Market Exit
Cigna (2)	7,657	6,725	Not in APCD
Colorado Choice Health Plans (3)	6,561	5,128	
Colorado HealthOP (1)	21,115	75,551	Not in APCD; 2016 Market Exit
Denver Health and Medical Plan (3)	840	580	In APCD in 2015 but not 2014
Humana (2)(4)	9,630	6,472	Not in APCD
Kaiser Permanente	76,523	58,654	
Rocky Mountain Health Plans (HMO) (2)(4)	26,695	22,630	Not in APCD
UnitedHealthcare (3)	254	194	
**Total Enrollment**	**193,065**	**217,795**	
Cumulative Total Covered Lives[5]	125,006	157,831	

© 2021 Bhanja A et al. *JAMA Health Forum*.

Headcount without FPL Merge	141,799	145,513	
Headcount with FPL Merge	62,336	64,165	

(1) Never submitted to the APCD; Left market

(2) Submitting to APCD - Not appropriately submitting Exchange membership

(3) Confirming status of significant variance

(4) Confirmed and resubmitting data

Data and Study Design (continued)

For our analyses, we excluded individuals who qualified for Medicaid via disability[6] or pregnancy-related eligibility due to differences in income eligibility criteria. We also excluded marketplace individuals who had incomes below 138% of FPL because this group consists primarily of individuals who are ineligible for Medicaid by immigration status.[7]

Regression Discontinuity Equations

For our outcome, enrollment during the open enrollment period, $Enrollment_i$, where i is any subgroup of enrollment (i.e. year, age, sex, etc.), we used the following regression discontinuity equations. The treatment effect of Marketplace eligibility vs. Medicaid eligibility is captured by the coefficient, τ, expressed as an incident rate ratio and calculated as percent change in enrollment below and above our threshold. $Enrollment_i$ was defined by the total persons enrolled in coverage by year and by age, sex, chronic condition status or urban-rural status, in our data. D is a binary indicator for whether a person's income was above the Medicaid threshold of 138% of FPL or not. The Beta coefficients control for the linear (or quadratic and cubic, depending on the model) relationship between income and enrollment. All equations used a generalized linear model with a negative binomial distribution and log link.

Linear Interaction Model

$$Enrollment_i = \alpha + \tau D + \beta_0(FPL - Threshold) + \beta_1 D(FPL - Threshold) + \varepsilon$$
$$\text{where } D = \begin{bmatrix} 0 & if\ FPL \leq Threshold \\ 1 & if\ FPL > Threshold \end{bmatrix}$$
$$and\ Threshold = 138$$

Quadratic Interaction Model

$$Enrollment_i = \alpha + \tau D + \beta_0(FPL - Threshold) + \beta_1 D(FPL - Threshold) + \beta_2(FPL - Threshold)^2 + \beta_3 D(FPL - Threshold)^2 + \varepsilon$$
$$\text{where } D = \begin{bmatrix} 0 & if\ FPL \leq Threshold \\ 1 & if\ FPL > Threshold \end{bmatrix}$$
$$and\ Threshold = 138$$

Cubic Interaction Model

$$Enrollment_i = \alpha + \tau D + \beta_0(FPL - Threshold) + \beta_1 D(FPL - Threshold) + \beta_2(FPL - Threshold)^2 + \beta_3 D(FPL - Threshold)^2 + \beta_4(FPL - Threshold)^3 + \beta_5 D(FPL - Threshold)^3 + \varepsilon$$
$$\text{where } D = \begin{bmatrix} 0 & if\ FPL \leq Threshold \\ 1 & if\ FPL > Threshold \end{bmatrix}$$
$$and\ Threshold = 138$$

Local Linear Model (Non-parametric)

$$Enrollment_i = \alpha + \tau D + \beta_0(FPL - Threshold) + \beta_1 D(FPL - Threshold) + \varepsilon$$
$$\text{where } D = \begin{bmatrix} 0 & if\ Threshold - Bandwidth < FPL \leq Threshold \\ 1 & if\ Threshold + Bandwidth \geq FPL > Threshold \end{bmatrix}$$
$$and\ Threshold = 138$$

$Bandwidth$ was set using the Stata SE 14 MSE‒optimal bandwidth selector for the RD treatment effect estimator e(h_mserd), defined by the command rdbwselect using the 2014 enrollment outcome, (*Bandwidth = 18)*.[8, 9]

Sensitivity Analyses

To confirm our findings with methods that are more typical of regression discontinuity designs, we replicated our models using a linear regression on enrollment in each bin of income (a percentage-point of FPL). We presented the

© 2021 Bhanja A et al. *JAMA Health Forum*.

regression coefficients for both parametric and non-parametric approaches, with which calculated the relative drop-off in enrollment at the eligibility threshold as a percentage of average enrollment between 133-138% FPL. The results of these analyses proved to be consistent with our primary findings.

Since our ACPD sample does not capture population denominators for each FPL (i.e. people not enrolled in Medicaid or Marketplace coverage), we also examined the income distribution of the total population, using the American Community Survey (ACS). We repeated our main model for the total population count above and below the 138% FPL cutoff, with family income defined based on the notion of the health insurance unit – an adult, spouse, and dependent children, excluding other adult relatives or unrelated roommates.

References:

1. Allen H, Gordon SH, Lee D, Bhanja A, Sommers BD. Comparison of Utilization, Costs, and Quality of Medicaid vs Subsidized Private Health Insurance for Low-Income Adults. JAMA Netw Open. 2021;4(1):e2032669. doi:10.1001/jamanetworkopen.2020.32669
2. van Walraven C, Austin PC, Jennings A, Quan H, Forster AJ. A modification of the Elixhauser comorbidity measures into a point system for hospital death using administrative data. Medical care. 2009:626-633.
3. Connect for Health Colorado. By the Numbers: The First Open Enrollment of Connect For Health Colorado. 2014. https://connectforhealthco.com/wp-content/uploads/2014/05/FINAL-data-open-enrollment-report-4-14-141.pdf
4. Connect for Health Colorado. By the Numbers: Colorado's Second Open Enrollment. 2015. https://connectforhealthco.com/wp-content/uploads/2015/03/2014-OE2-Report.pdf
5. Connect for Health Colorado. Marketplace Dashboard. 2014. https://connectforhealthco.com/c4-media/wp-content/uploads/2014/10/Marketplace-Dashboard-10-01-2014.pdf.
6. Social Security Administration. *Annual Statistical Supplement to the Social Security Bulletin*. 2014:20. https://www.ssa.gov/policy/docs/statcomps/supplement/2014/supplement14.pdf
7. U.S. Center for Medicaid and Medicare Services. Coverage for lawfully present immigrants. https://www.healthcare.gov/immigrants/lawfully-present-immigrants/
8. Imbens G, Kalyanaraman K. Optimal bandwidth choice for the regression discontinuity estimator. The Review of economic studies. 2012;79(3):933-959.
9. Calonico S, Cattaneo MD, Titiunik R. Robust nonparametric confidence intervals for regression-discontinuity designs. Econometrica. 2014;82(6):2295-2326.

© 2021 Bhanja A et al. *JAMA Health Forum*.

eFigure 1. Number of Medicaid/Marketplace Enrollees By Gender and Income, 75-400%FPL, 2014 & 2015

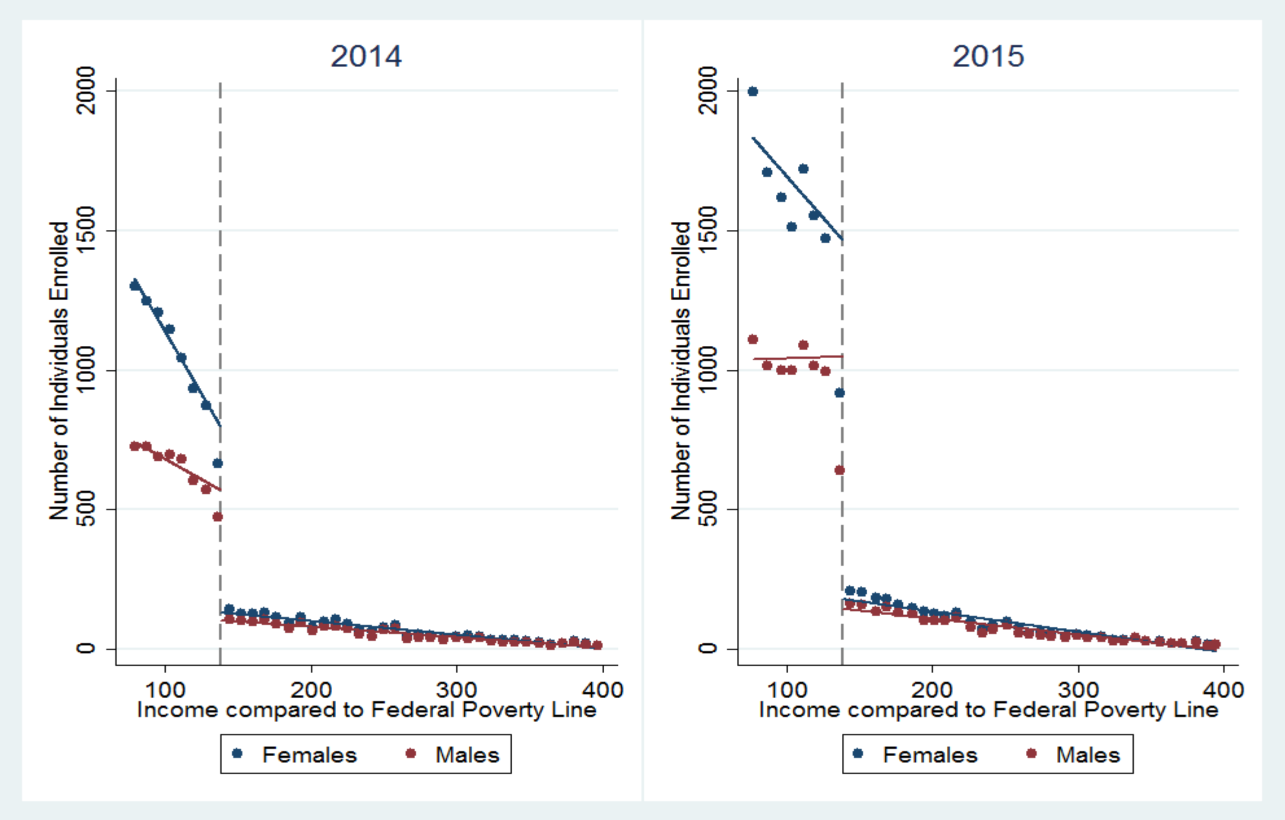

Source: Authors analysis of Colorado all payer claims database from 2014-2015
Notes: Sample is limited to non-disabled and non-pregnant adults aged 19-64 whose Medicaid or Marketplace coverage became active during the ACA's open enrollment period (January 1-April 15, 2014 and January 1-March 3, 2015).

© 2021 Bhanja A et al. *JAMA Health Forum*.

eFigure 2. Number of Medicaid/Marketplace Enrollees By Residence, Urban vs. Rural, and Income, 75-400%FPL, 2014 & 2015

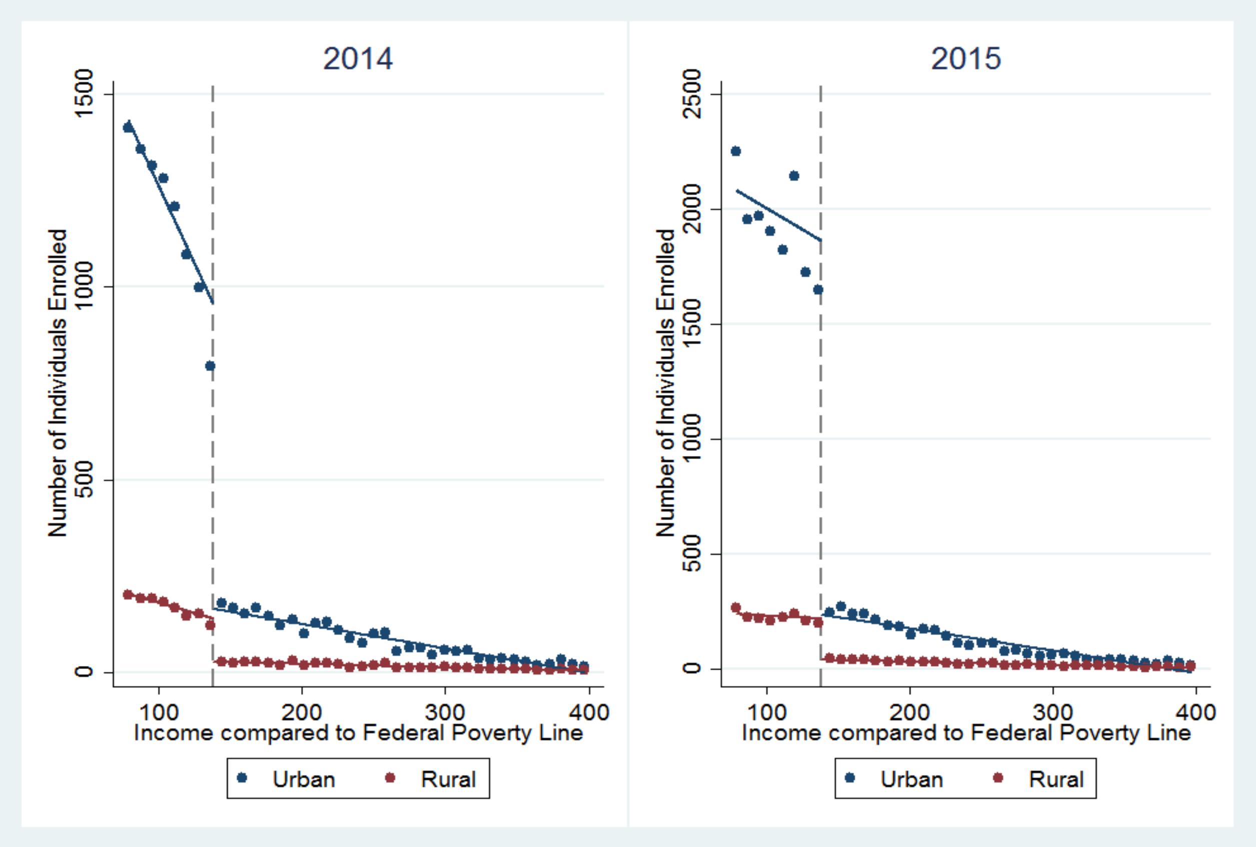

Source: Authors analysis of Colorado all payer claims database from 2014-2015
Notes: Sample is limited to non-disabled and non-pregnant adults aged 19-64 whose Medicaid or Marketplace coverage became active during the ACA's open enrollment period (January 1-April 15, 2014 and January 1-March 3, 2015).

© 2021 Bhanja A et al. *JAMA Health Forum*.

eFigure 3. Number of Medicaid/Marketplace Enrollees By Chronic Condition Status and Income, 75-400%FPL, 2014 & 2015

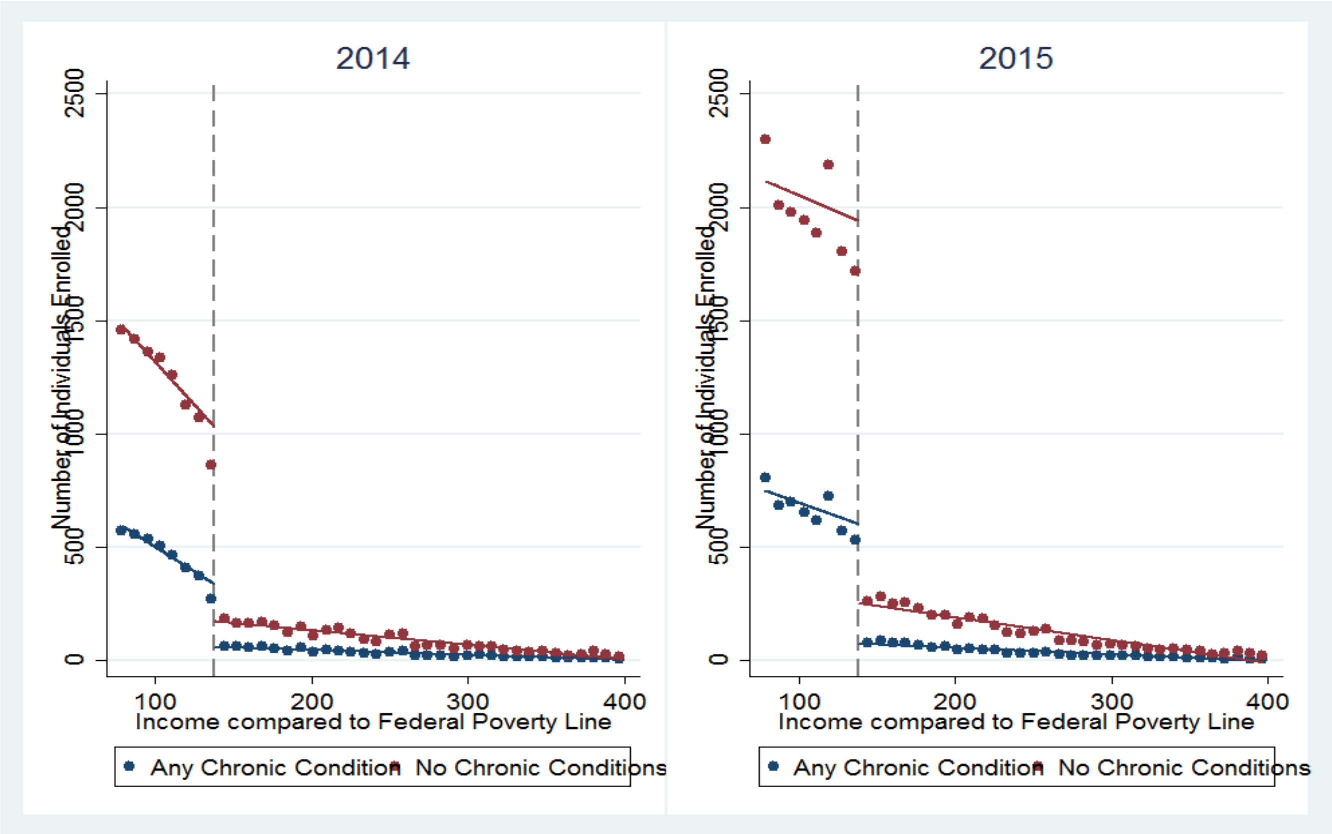

Source: Authors analysis of Colorado all payer claims database from 2014-2015

Notes: Sample is limited to non-disabled and non-pregnant adults aged 19-64 whose Medicaid or Marketplace coverage became active during the ACA's open enrollment period (January 1-April 15, 2014 and January 1-March 3, 2015).

© 2021 Bhanja A et al. *JAMA Health Forum*.

eTable 2. Open Enrollment, Full Linear, Quadratic, Cubic, and Local Linear Regression Discontinuity

	Average N at 133-138 FPL		Full Linear		Full Quadratic		Full Cubic		Local Linear	
	2014	2015	2014	2015	2014	2015	2014	2015	2014	2015
**Open Enrollment**										
**RD coefficient at Threshold**	1424 (353.33)	2882 (383.83)	0.211	0.167	0.194	0.142	0.183	0.148	0.187	0.114
Standard Error			0.059	0.046	0.079	0.027	0.099	0.079	0.023	0.012
p-value			<0.001	<0.001	<0.001	<0.001	<0.001	<0.001	<0.001	<0.001
N			326	326	326	326	326	326	326	326
RELATIVE ENROLLMENT DROP-OFF			**-78.9**	**-83.3**	**-80.6**	**-85.8**	**-81.7**	**-85.2**	**-81.3**	**-88.6**

Source: Authors analysis of Colorado all payer claims database from 2014-2015

© 2021 Bhanja A et al. *JAMA Health Forum*.

eTable 3. Open Enrollment by Age, Full Linear, Quadratic, Cubic, and Local Linear Regression Discontinuity, 2014

		Full Linear		Full Quadratic		Full Cubic		Local Linear	
	Average N between 133-138% FPL (s.d.)		Between Group p-value		Between Group p-value		Between Group p-value		Between Group p-value
**Age**									
**19-25**									
Coefficient at Threshold	211 (74.96)	0.113	<0.001	0.119	<0.001	0.118	<0.001	0.133	<0.001
Standard Error		0.011		0.017		0.023		0.028	
p-value		<0.001		<0.001		<0.001		<0.001	
RELATIVE ENROLLMENT DROP-OFF		**-88.7**		**-88.1**		**-88.2**		**-86.7**	
**26-34**									
Coefficient at Threshold	432 (146.56)	0.171	<0.001	0.146	<0.001	0.116	<0.001	0.113	<0.001
Standard Error		0.019		0.023		0.023		0.029	
p-value		<0.001		<0.001		<0.001		<0.001	
RELATIVE ENROLLMENT DROP-OFF		**-82.9**		**-85.4**		**-88.4**		**-88.7**	
**35-44**									
Coefficient at Threshold	332 (63.04)	0.163	<0.001	0.147	<0.001	0.129	<0.001	0.122	<0.001
Standard Error		0.012		0.014		0.016		0.017	
p-value		<0.001		<0.001		<0.001		<0.001	
RELATIVE ENROLLMENT DROP-OFF		**-83.7**		**-85.3**		**-87.1**		**-87.8**	
**45-54**									
Coefficient at Threshold	245 (40.86)	0.263	<0.001	0.243	<0.001	0.243	<0.001	0.254	<0.001
Standard Error		0.02		0.024		0.03		0.028	
p-value		<0.001		<0.001		<0.001		<0.001	
RELATIVE ENROLLMENT DROP-OFF		**-73.7**		**-75.7**		**-75.7**		**-74.6**	
**55-64**									
Coefficient at Threshold	204 (61.62)	0.436	ref	0.389	ref	0.41	ref	0.436	ref
Standard Error		0.043		0.054		0.076		0.081	
p-value		<0.001		<0.001		<0.001		<0.001	
RELATIVE ENROLLMENT DROP-OFF		**-56.4**		**-61.1**		**-59.0**		**-56.4**	

Source: Authors analysis of Colorado all payer claims database from 2014-2015

© 2021 Bhanja A et al. *JAMA Health Forum*.

Notes: p-values calculated from chi-square and adjusted Wald tests (Local Linear only)

© 2021 Bhanja A et al. *JAMA Health Forum*.

eTable 4. Open Enrollment by Age, Full Linear, Quadratic, Cubic, and Local Linear Regression Discontinuity, 2015

		**Full Linear**		**Full Quadratic**		**Full Cubic**		**Local Linear**	
	**Average N between 133-138% FPL (s.d.)**		**Between Group p-value**		**Between Group p-value**		**Between Group p-value**		**Between Group p-value**
**Age**									
**19-25**									
Coefficient at Threshold	422 (73.94)	0.119	<0.001	0.121	<0.001	0.127	<0.001	0.114	<0.001
Standard Error		0.009		0.011		0.015		0.011	
p-value		<0.001		<0.001		<0.001		<0.001	
RELATIVE ENROLLMENT DROP-OFF		**-88.1**		**-87.9**		**-87.3**		**-88.6**	
**26-34**									
Coefficient at Threshold	869 (105.60)	0.146	<0.001	0.128	<0.001	0.117	<0.001	0.081	<0.001
Standard Error		0.011		0.013		0.015		0.016	
p-value		<0.001		<0.001		<0.001		<0.001	
RELATIVE ENROLLMENT DROP-OFF		**-85.4**		**-87.2**		**-88.3**		**-91.9**	
**35-44**									
Coefficient at Threshold	711 (100.94)	0.134	<0.001	0.105	<0.001	0.113	<0.001	0.07	<0.001
Standard Error		0.008		0.008		0.013		0.009	
p-value		<0.001		<0.001		<0.001		<0.001	
RELATIVE ENROLLMENT DROP-OFF		**-86.6**		**-89.5**		**-88.7**		**-93.0**	
**45-54**									
Coefficient at Threshold	496 (68.53)	0.197	<0.001	0.157	<0.001	0.163	<0.001	0.134	<0.001
Standard Error		0.013		0.013		0.018		0.014	
p-value		<0.001		<0.001		<0.001		<0.001	
RELATIVE ENROLLMENT DROP-OFF		**-80.3**		**-84.3**		**-83.7**		**-86.6**	
**55-64**									
Coefficient at Threshold	385 (83.53)	0.308	ref	0.27	ref	0.286	ref	0.247	ref
Standard Error		0.024		0.028		0.041		0.036	
p-value		<0.001		<0.001		<0.001		<0.001	
RELATIVE ENROLLMENT DROP-OFF		**-69.2**		**-73.0**		**-71.4**		**-75.3**	

Source: Authors analysis of Colorado all payer claims database from 2014-2015

© 2021 Bhanja A et al. *JAMA Health Forum*.

Notes: p-values calculated from chi-square and adjusted Wald tests (Local Linear only)

© 2021 Bhanja A et al. *JAMA Health Forum*.

eTable 5. Open Enrollment by Sex, Chronic Condition and Residence, Full Linear, Quadratic, Cubic, and Local Linear Regression Discontinuity, 2014

		Full Linear		Full Quadratic		Full Cubic		Local Linear	
	Average N between 133-138% FPL (s.d.)		Between Group p-value		Between Group p-value		Between Group p-value		Between Group p-value
**Sex**									
**Female**									
Coefficient at Threshold	835 (166.61)	0.202	0.004	0.184	0.03	0.176	0.18	0.184	0.57
Standard Error		0.015		0.019		0.023		0.023	
p-value		<0.001		<0.001		<0.001		<0.001	
RELATIVE ENROLLMENT DROP-OFF		**-79.8**		**-81.6**		**-82.4**		**-81.6**	
**Male**									
Coefficient at Threshold	589 (191.71)	0.222	ref	0.207	ref	0.194	ref	0.193	ref
Standard Error		0.02		0.027		0.034		0.035	
p-value		<0.001		<0.001		<0.001		<0.001	
RELATIVE ENROLLMENT DROP-OFF		**-77.8**		**-79.3**		**-80.6**		**-80.7**	
**Chronic Condition**									
**Any Condition**									
Coefficient at Threshold	338 (84.00)	0.212	0.81	0.213	0.02	0.219	0.002	0.207	0.14
Standard Error		0.017		0.024		0.033		0.032	
p-value		<0.001		<0.001		<0.001		<0.001	
RELATIVE ENROLLMENT DROP-OFF		**-78.8**		**-78.7**		**-78.1**		**-79.3**	
**No condition**									
Coefficient at Threshold	1086 (279.33)	0.21	ref	0.188	ref	0.173	ref	0.181	ref
Standard Error		0.018		0.022		0.026		0.027	
p-value		<0.001		<0.001		<0.001		<0.001	
RELATIVE ENROLLMENT DROP-OFF		**-79.0**		**-81.2**		**-82.7**		**-81.9**	
**Residence**									
**Urban**									
Coefficient at Threshold	999 (247.30)	0.223	0.98	0.196	0.81	0.19	0.98	0.187	0.24
Standard Error		0.018		0.022		0.028		0.026	
p-value		<0.001		<0.001		<0.001		<0.001	
RELATIVE ENROLLMENT DROP-OFF		**-77.7**		**-80.4**		**-81.0**		**-81.3**	
**Rural**									

© 2021 Bhanja A et al. *JAMA Health Forum*.

Coefficient at Threshold	149 (38.98)	0.224	ref	0.199	ref	0.19	ref	0.21	ref
Standard Error		0.021		0.026		0.033		0.035	
p-value		<0.001		<0.001		<0.001		<0.001	
RELATIVE ENROLLMENT DROP-OFF		**-77.6**		**-80.1**		**-81.0**		**-82.0**	

Source: Authors analysis of Colorado all payer claims database from 2014-2015 **Notes:** p-values calculated from chi-square and adjusted Wald tests (Local Linear only)

© 2021 Bhanja A et al. *JAMA Health Forum*.

eTable 6. Open Enrollment by Sex, Chronic Condition and Residence, Full Linear, Quadratic, Cubic, and Local Linear Regression Discontinuity, 2015

		**Full Linear**		**Full Quadratic**		**Full Cubic**		**Local Linear**	
	**Average N between 133-138% FPL (s.d.)**		**Between Group p-value**		**Between Group p-value**		**Between Group p-value**		**Between Group p-value**
**Sex**									
**Female**									
Coefficient at Threshold	1693 (218.08)	0.159	<0.001	0.134	<0.001	0.142	0.02	0.105	<0.001
Standard Error		0.01		0.01		0.015		0.012	
p-value		<0.001		<0.001		<0.001		<0.001	
RELATIVE ENROLLMENT DROP-OFF		**-84.1**		**-86.6**		**-85.8**		**-89.5**	
**Male**									
Coefficient at Threshold	1187 (176.86)	0.177	ref	0.155	ref	0.157	ref	0.127	ref
Standard Error		0.012		0.013		0.018		0.014	
p-value		<0.001		<0.001		<0.001		<0.001	
RELATIVE ENROLLMENT DROP-OFF		**-82.3**		**-84.5**		**-84.3**		**-87.3**	
**Chronic Condition**									
**Any Condition**									
Coefficient at Threshold	680 (107.75)	0.155	<0.001	0.142	0.99	0.154	0.27	0.115	0.65
Standard Error		0.01		0.012		0.018		0.013	
p-value		<0.001		<0.001		<0.001		<0.001	
RELATIVE ENROLLMENT DROP-OFF		**-84.5**		**-85.8**		**-84.6**		**-88.5**	
**No condition**									
Coefficient at Threshold	2202 (278.68)	0.171	ref	0.142	ref	0.147	ref	0.113	ref
Standard Error		0.011		0.011		0.015		0.012	
p-value		<0.001		<0.001		<0.001		<0.001	
RELATIVE ENROLLMENT DROP-OFF		**-82.9**		**-85.8**		**-85.3**		**-88.7**	
**Residence**									
**Urban**									
Coefficient at Threshold	2114 (259.76)	0.172	<0.001	0.142	<0.001	0.147	<0.001	0.109	0.016
Standard Error		0.012		0.012		0.016		0.013	
p-value		<0.001		<0.001		<0.001		<0.001	
RELATIVE ENROLLMENT DROP-OFF		**-82.8**		**-85.8**		**-85.3**		**-89.1**	
**Rural**									

© 2021 Bhanja A et al. *JAMA Health Forum*.

Coefficient at Threshold	248 (44.26)	0.204	ref	0.18	ref	0.199	ref	0.169	ref
Standard Error		0.015		0.018		0.027		0.026	
p-value		<0.001		<0.001		<0.001		<0.001	
RELATIVE ENROLLMENT DROP-OFF		**-79.6**		**-82.0**		**-80.1**		**-83.1**	

Source: Authors analysis of Colorado all payer claims database from 2014-2015 **Notes:** p-values calculated from chi-square and adjusted Wald tests (Local Linear only)

© 2021 Bhanja A et al. *JAMA Health Forum*.

eTable 7. Open Enrollment, Full Linear, Quadratic Cubic, and Local Linear Regression Discontinuity Sensitivity Analysis

	Average N at 133-138% FPL		Full Linear		Full Quadratic		Full Cubic		Local Linear	
	2014	2015	2014	2015	2014	2015	2014	2015	2014	2015
**Open Enrollment**										
**RD coefficient at Threshold**	1424 (353.33)	2882 (383.83)	-1137.106	-2217.138	-1044.484	-2405.840	-1117.603	-2184.223	-1087.474	-2441.218
Standard Error			58.19)	120.94	99.22	143.16	147.60	212.69	166.04	214.53
p-value			<0.001	<0.001	<0.001	<0.001	<0.001	<0.001	<0.001	<0.001
N			326	326	326	326	326	326	37	37
RELATIVE ENROLLMENT DROP-OFF			**-79.9**	**-76.9**	**-73.4**	**-83.5**	**-78.5**	**-75.8**	**-76.4**	**-84.7**

Source: Authors analysis of Colorado all payer claims database from 2014-2015

© 2021 Bhanja A et al. *JAMA Health Forum*.

eFigure 4. Population by Income, Colorado American Community Survey, 2014 -2015

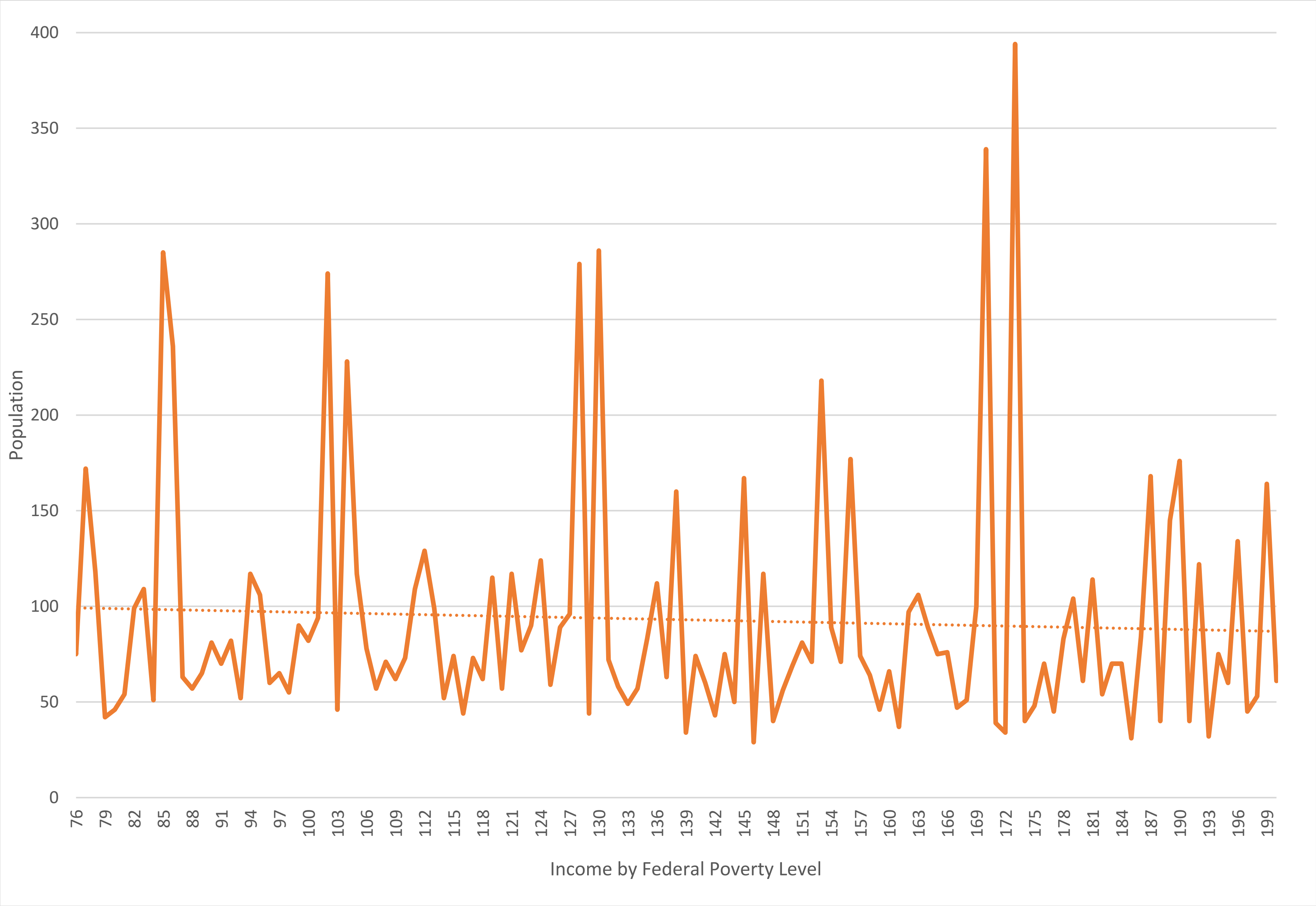

Source: Authors analysis of American Community Survey data, Colorado 2014-2015

© 2021 Bhanja A et al. *JAMA Health Forum*.
